# Supplementary material for: Venomix: a simple bioinformatic pipeline for identifying and characterizing toxin gene candidates from transcriptomic data
Source: PeerJ. 2018 Jul 31;6:e5361. doi: 10.7717/peerj.5361 (PMC6074769; doi:10.7717/peerj.5361)
Supplement: Supplemental Information 6 [file peerj-06-5361-s006.gz › FinalOutput_1E-6/Dortoxin_8/finaltree.pdf]

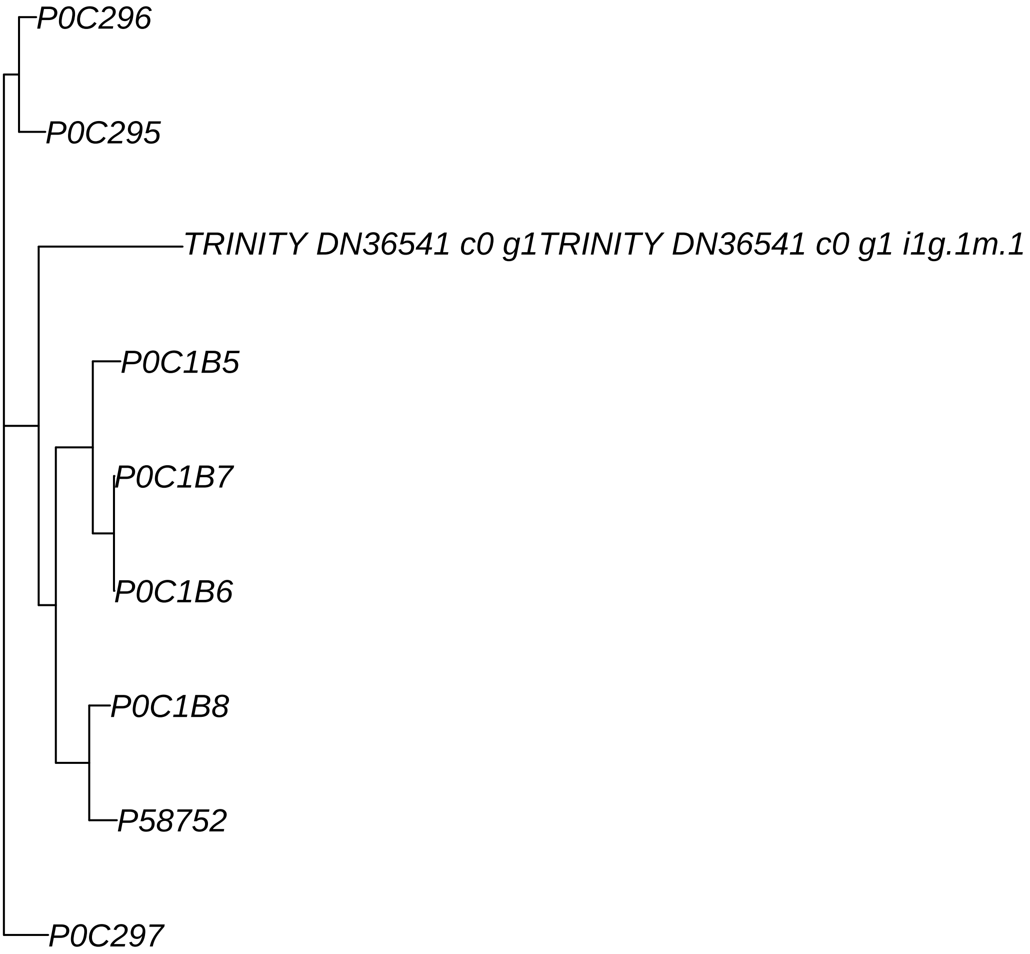
P0C296
P0C295
TRINITY DN36541 c0 g1TRINITY DN36541 c0 g1 i1g.1m.1
P0C1B5
P0C1B7
P0C1B6
P0C1B8
P58752
P0C297
